# Whole-genome resequencing of *Coffea arabica* L. (Rubiaceae) genotypes identify SNP and unravels distinct groups showing a strong geographical pattern

**DOI:** 10.1186/s12870-022-03449-4

**Published:** 2022-02-14

**Authors:** Yeshitila Mekbib, Kassahun Tesfaye, Xiang Dong, Josphat K. Saina, Guang-Wan Hu, Qing-Feng Wang

**Affiliations:** 1 CAS Key Laboratory of Plant Germplasm Enhancement and Specialty Agriculture, Wuhan Botanical Garden, Chinese Academy of Sciences, Wuhan, 430074 China; 2 Ethiopian Biodiversity Institute, P.O. Box 30726, Addis Ababa, Ethiopia; 3 University of Chinese Academy of Sciences, Beijing, 100049 China; 4 Sino-Africa Joint Research Center, Chinese Academy of Sciences, Wuhan, 430074 China; 5 Department of Microbial, Cellular and Molecular Biology, Addis Ababa University, Addis Ababa, Ethiopia; 6 Ethiopian Biotechnology Institute, Ministry of Innovation and Technology, Addis Ababa, Ethiopia; 7 Centre for Integrative Conservation, Xishuangbanna Tropical Botanical Garden, Chinese Academy of Sciences, Menglun, 666303 China

**Keywords:** Coffee, Genetic markers, Phylogenetic analysis, Resequencing, Single nucleotide polymorphism

## Abstract

**Background:**

*Coffea arabica* L. is an economically important agricultural crop and the most popular beverage worldwide. As a perennial crop with recalcitrant seed, conservation of the genetic resources of coffee can be achieved through the complementary approach of in-situ and ex-situ field genebank. In Ethiopia, a large collection of *C. arabica* L. germplasm is preserved in field gene banks. Here, we report the whole-genome resequencing of 90 accessions from Choche germplasm bank representing garden and forest-based coffee production systems using Illumina sequencing technology.

**Results:**

The genome sequencing generated 6.41 billion paired-end reads, with a mean of 71.19 million reads per sample. More than 93% of the clean reads were mapped onto the *C. arabica* L. reference genome. A total of 11.08 million variants were identified, among which 9.74 million (87.9%) were SNPs (Single nucleotide polymorphisms) and 1.34 million (12.1%) were InDels. In all accessions, genomic variants were unevenly distributed across the coffee genome. The phylogenetic analysis using the SNP markers displayed distinct groups.

**Conclusions:**

Resequencing of the coffee accessions has allowed identification of genetic markers, such as SNPs and InDels. The SNPs discovered in this study might contribute to the variation in important pathways of genes for important agronomic traits such as caffeine content, yield, disease, and pest in coffee. Moreover, the genome resequencing data and the genetic markers identified from 90 accessions provide insight into the genetic variation of the coffee germplasm and facilitate a broad range of genetic studies.

**Supplementary Information:**

The online version contains supplementary material available at 10.1186/s12870-022-03449-4.

## Introduction

Coffee (Rubiaceae) is an important agricultural crop and is mainly grown as a cash crop in several tropical countries [1, 2]. Presently, it is cultivated in more than 80 countries around the globe [3] and serves as a major source of livelihood for smallholder farmers. With only 30% of production consumed domestically, coffee remains an important export commodity [4]. Despite the huge number of species reported in the genus *Coffea* [5], the primary species utilized for coffee production are *Coffea arabica* L. and *C. canephora* Pierre [2, 6]. Arabica coffee is a tetraploid species (2n = 4x = 44) derived from interspecific crosses between *C. canephora* and *C. eugenioides* [7]. The cultivation of this crop serves as an important source of income and employment in developing countries of Latin America, Africa, and Asia [8]. Besides, *C. arabica* L. is valued for its superior beverage quality [9–11] and accounts for about 63% of the global coffee production [11].

*Coffea arabica* L. is the only coffee species cultivated and exported from Ethiopia [12]. It is the major foreign exchange earner contributing to a quarter of the country’s export earnings [13] and serves as a means of livelihood and employment for an estimated 15 million people [12–14]. The agro-ecology under which coffee grows varies significantly, and the crop is mainly produced in four distinct production systems in Ethiopia; i.e., garden, semi-forest, forest and plantation. Garden coffee is widespread across the country and forest-based (semi-forest and forest) coffee production systems are found in the southeast and southwest parts of the country [14]. Different scholars have reported a high genetic diversity for coffee in Ethiopia, which is of great potential to improve the crop [15–18]. Presently, the global demand for specialty coffee has increased significantly [19]. Hence, developing coffee varieties with high market demand, such as low bean caffeine content, is essential. Ethiopia being the main source of *C. arabica* L. gene pool, the germplasm found in the country is valuable [16], and could be used for developing varieties with desirable traits [20–22]. Notably, the wild coffee genetic resources are genetically diverse and are believed to possess traits that can be used to improve the cultivated varieties [17, 23]. Specifically, these resources are valuable in light of the projected climate change due to their ability to adapt to environmental change [24]. Despite this fact, the forest coffee gene pool of *C. arabica* L. is threatened by various factors that could affect the genetic base for the future breeding program [25]. The loss is mainly attributed to deforestation and land-use change [15], climate change [13, 26], and the introduction of new varieties in the coffee forests [27].

Presently, global ex-situ coffee germplasm conservation programs have been implemented in various countries including Ethiopia [28]. Coffee germplasm is conserved as a living tree in the field gene bank due to the recalcitrant nature of the seeds [29]. Likewise, in Ethiopia, the long-term preservation of this important cash crop is achieved by establishing field gene banks. Currently, more than 11,000 coffee accessions collected by random and non-random sampling techniques are maintained in field gene banks in Ethiopia [30]. Evaluating the genetic diversity of the coffee germplasm maintained in field genebank is essential [31], and assists in enhancing the management of the conserved materials. However, the perennial nature of coffee makes the evaluation and breeding work very costly [32]. Hence, developing and using molecular markers in coffee could enhance the development of varieties with desirable traits [33].

Currently, NGS technology has enabled the identification of genetic variation in germplasm collections [34]. Particularly, the availability of reference genome and improvement in the genetic data analysis methods have contributed to advance resequencing studies [35, 36]. Genome resequencing could also help in bridging the knowledge gap between genotype and phenotype and facilitate molecular breeding [37]. Notably, the markers generated from resequencing analysis help to advance the conventional crop breeding approaches [38], and in turn, contribute to shortening the time required to develop new varieties [39].

SNP represent single nucleotide change in DNA sequence and are considered the most abundant form of genetic variation [37, 40]. Presently, SNPs have become the genetic markers of choice in various genetic, ecological and evolutionary studies [41]. Despite the great economic and social importance of *C. arabica* L., studies with SNP markers are scarce and a small number of SNP markers are available for this species [33]. This study, therefore, aimed to discover the genomic variations in 90 accessions of *C. arabica* L. by whole-genome resequencing. The genomic data and SNP generated in this study could be of great relevance for undertaking various genetic studies.

## Materials and methods

### Sample preparation and DNA extraction

The *C. arabica* L. accessions that have been maintained at the Choche germplasm bank of the Ethiopian Biodiversity Institute (Jimma zone, Goma district, southwest Ethiopia) were used in this study. Genomic DNA was isolated from Silica gel dried leaf material of the 90 accessions originally collected from the garden and forest-based coffee production systems using MagicMag Genomic DNA Micro Kit (Sangon Biotech Co. Shanghai, China). The quantity and quality of isolated DNA were checked and analyzed with the NanoDrop2000 spectrophotometer (Thermo Fisher Scientific, Waltham, MA, USA) and 1.0% agarose gel electrophoresis, respectively. Detailed information about each accession included in this study is shown in Additional file 1: Table S1.

### Library preparation and sequencing

Paired-end libraries with approximately 350 bp insert sizes were constructed from 1 μg of genomic DNA from each accession using Illumina TruSeq or Nextera (San Diego, CA) kits according to the Illumina manufacturer’s specifications. Whole-genome resequencing was performed for 90 coffee accessions at the Beijing Genomics Institute (Shenzhen, China) using the Illumina Hiseq 2500 Platform (Illumina, San Diego, CA).

### Sequence processing and mapping of reads to the reference genome

The filtering of raw reads was accomplished using the FASTQC (version 0.11.3) program. The clean reads were aligned onto the *C. arabica* L. reference genome using a burrows wheeler aligner (BWA) with default parameters [42]. SAMtools (version 1.3.1) software was used to convert mapping results into the BAM format and filter the unmapped reads [43]. Then, the aligned reads were processed using Piccard tools (http://broadinstitute.github.io/picard/) to remove duplicate reads. The Illumina sequencing reads of each accession is deposited under accession number from SRR17316330 to SRR17316419.

### Variant detection, annotation and relationship analysis

The mapped reads were used to detect variants (SNP and InDels) using the Genome Analysis Toolkit (version 3.6) software [44]. The annotation and classification of the genomic variants were performed by SnpEff software [45]. The variants were annotated based on their impact (high, moderate, modifier and low), functional class (synonymous and non-synonymous substitutions) and their genomic regions such as downstream, upstream, exon, intron, intragenic and intergenic region, transcript, 3′ and 5′ untranslated regions (UTRs). DNA substitution mutations (transitions and transversion) and amino acids changes were identified. The whole-genome SNP markers were used to infer the phylogenetic relationship of 90 accessions. A Maximum likelihood (ML) analysis was conducted using the RAxML (version 8.1.2) program [46]. 

## Results

### Resequencing 90 accessions of *C. arabica* L.

Whole-genome resequencing of 90 accessions of *C. arabica* L. was performed with the Illumina sequencing platform. The genome sequencing generated 6.41billion paired-end reads, with a mean of 71.19 million reads per sample. After removing low-quality reads, high-quality reads of each accession were mapped onto the *C. arabica* L. reference genome using a BWA aligner. The percentage of reads mapped onto the reference genome varied from 88.93 to 98.11%. The detailed resequencing information was provided in Additional file 2: Table S2. This result indicates that there is a difference in the whole-genome sequences among the studied accessions. A total of 11.08 million variants were identified by mapping the clean reads onto the reference genome. Among these, 9.74 million (87.9%) were SNPs and 1.34 million (12.1%) were InDels (insertions and deletions) (Table 1). The deletions and insertions length observed in the coffee genome ranged from 1 to 46 base pairs.

**Table 1:** Type and the number of variants detected in the *C. arabica* L. genome

Variant	Total number	%
SNP	9,743,804	87.9
InDels		
- insertions	794,963	7.2
- deletions	545,345	4.9
Total	11,084,112	100

### Identification, characterization and annotation of SNPs

**Fig. 1:**
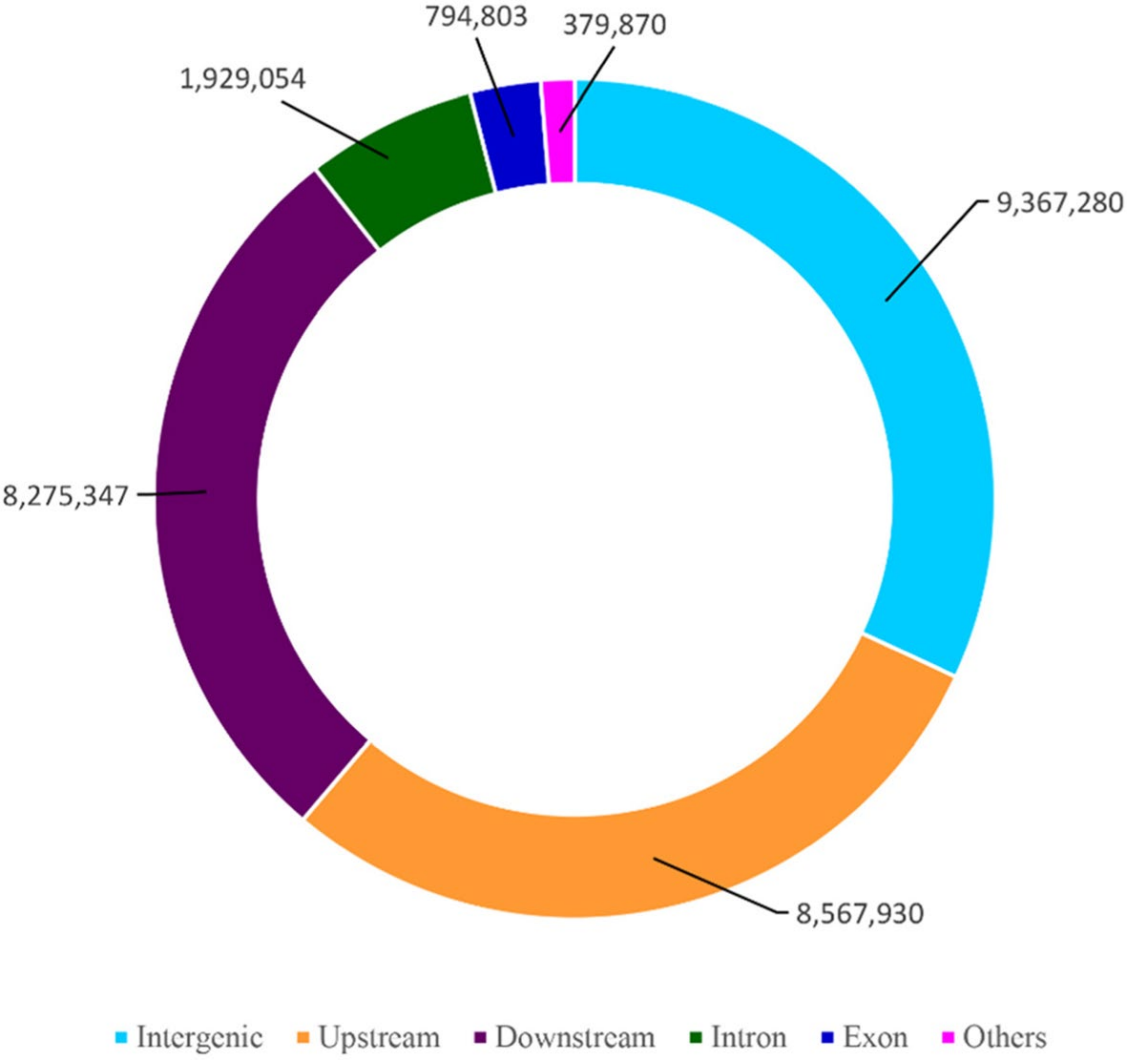
The distribution of the identified SNP in the different genomic regions (Intergenic, Upstream, Downstream, Intron, Exon and other) of the *C. arabica* L. genome

The high-quality sequences were used for the identification of SNP, and a total of 9.74 million SNPs were identified in all the 90 coffee accessions. We used the SnpEff [45] program to evaluate the impact and possible effects of the identified SNP could have on the gene. Based on their impact on the coding sequence the SNP were classified into four classes i.e., high, moderate, modifier and low. The analysis revealed a major proportion of SNPs were modifier (97.911% of the SNP with impact on non-coding regions), followed by moderate (1.151% of the SNP could have a non-synonymous substitution), low impact (0.834% of the SNP with synonymous substitution) and the smallest value was recorded for high impact SNP (0.104% of the SNP with disruptive impact on the protein) (Table 2). The distribution of SNPs across genomic regions was compared (Fig. 1). SNPs were most abundant in the intergenic, upstream, downstream, intron and exon of genes, and their proportions are about 29.115, 26.63, 25.721, 5.996 and 2.47%, respectively. A limited number of SNPs were observed in 3’UTR (0.574%) and 5’UTR (0.484). More SNPs were detected in the introns than exons (Fig. 1). Further, the result showed that the majority of genomic variations were located in non-coding regions. The SNP variants were also separated into heterozygous and homozygous, and in all coffee accessions, the number of heterozygous variants was higher than the homogenous ones. The SNP mutations also resulted in a codon modification in genomic regions leading to variations in amino acid sequences. The details of amino acid changes observed in this study are indicated in Additional file 3: Table S3.

**Table 2:** The effects of identified SNP on genes as classified by SnpEff program [45]

Impact class	Count	Percent
High	33,443	0.104%
Moderate	370,392	1.151%
Low	268,283	0.834%
Modifier	31,501,326	97.911%

**Fig. 2:**
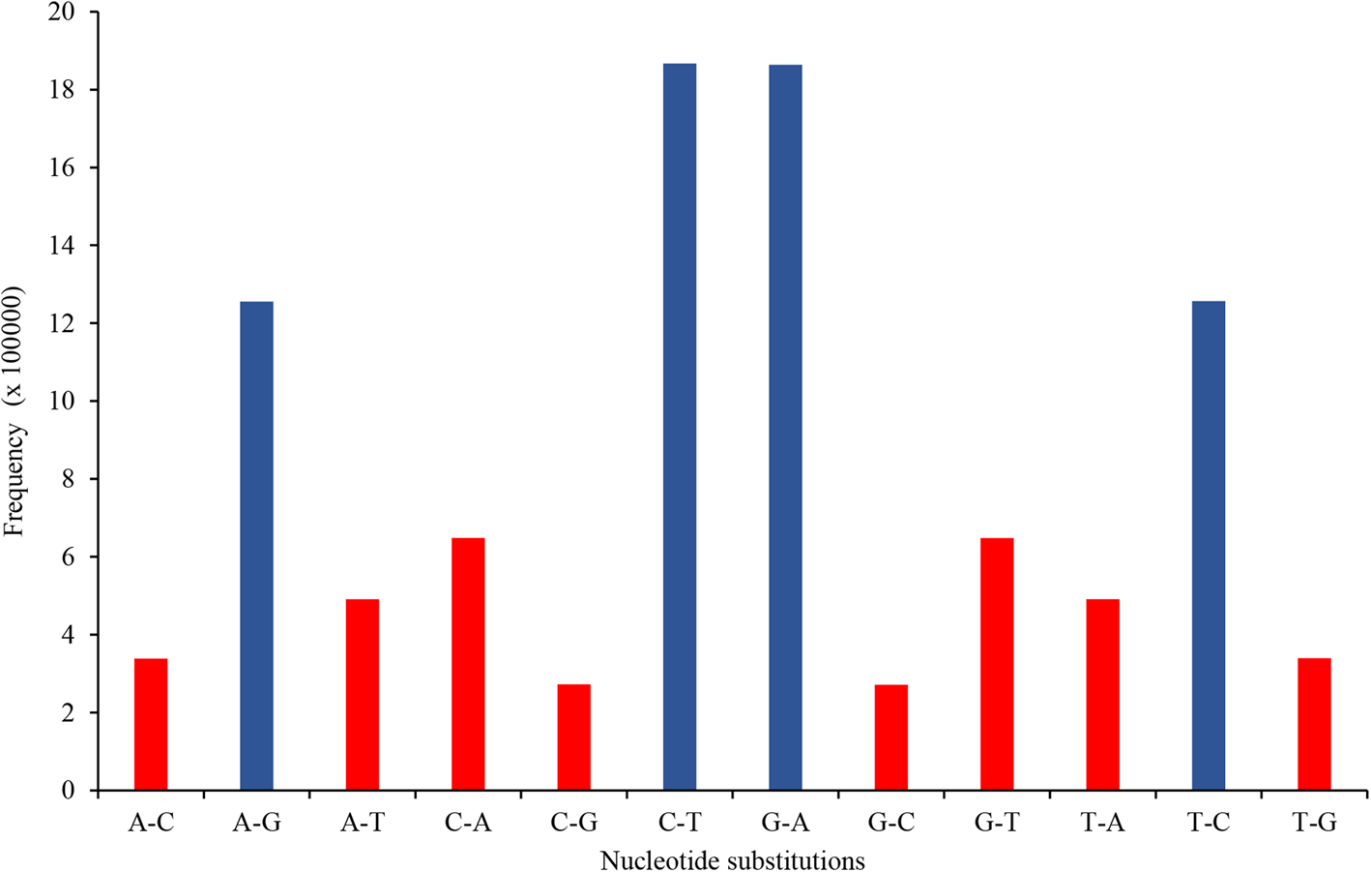
Transition and transversion SNPs detected in *C. arabica* L. genome (Transitions (A-G, C-T) indicated with red color; Transversions (C-G, A-C, G-T, A-T) indicated with blue color

Based on the nucleotide substitutions, the SNPs identified in the coffee genome were classified into two classes, namely, transitions (A/G and C/T) and transversions (A/C, C/G, A/T, G/T). The total transitions and transversions detected were 204,665,968 and 103,693,961, with a transitions/transversions (Ts/Tv) ratio of 1.97. The transition frequency of C/T was more than G/A. The transversion frequency of C/A was higher, similarly within transitions; the C/T transition was higher in number (Additional file 4: Table S4 and Fig. 2). The SNP found in the coding region are of two types i.e., synonymous and non-synonymous. A total of 215,712 synonymous SNPs were detected in the coding sequence of the coffee genome (Additional file 4: Table S4). Often, synonymous SNPs do not affect the normal function of genes.

### Relationship analysis

**Fig. 3:**
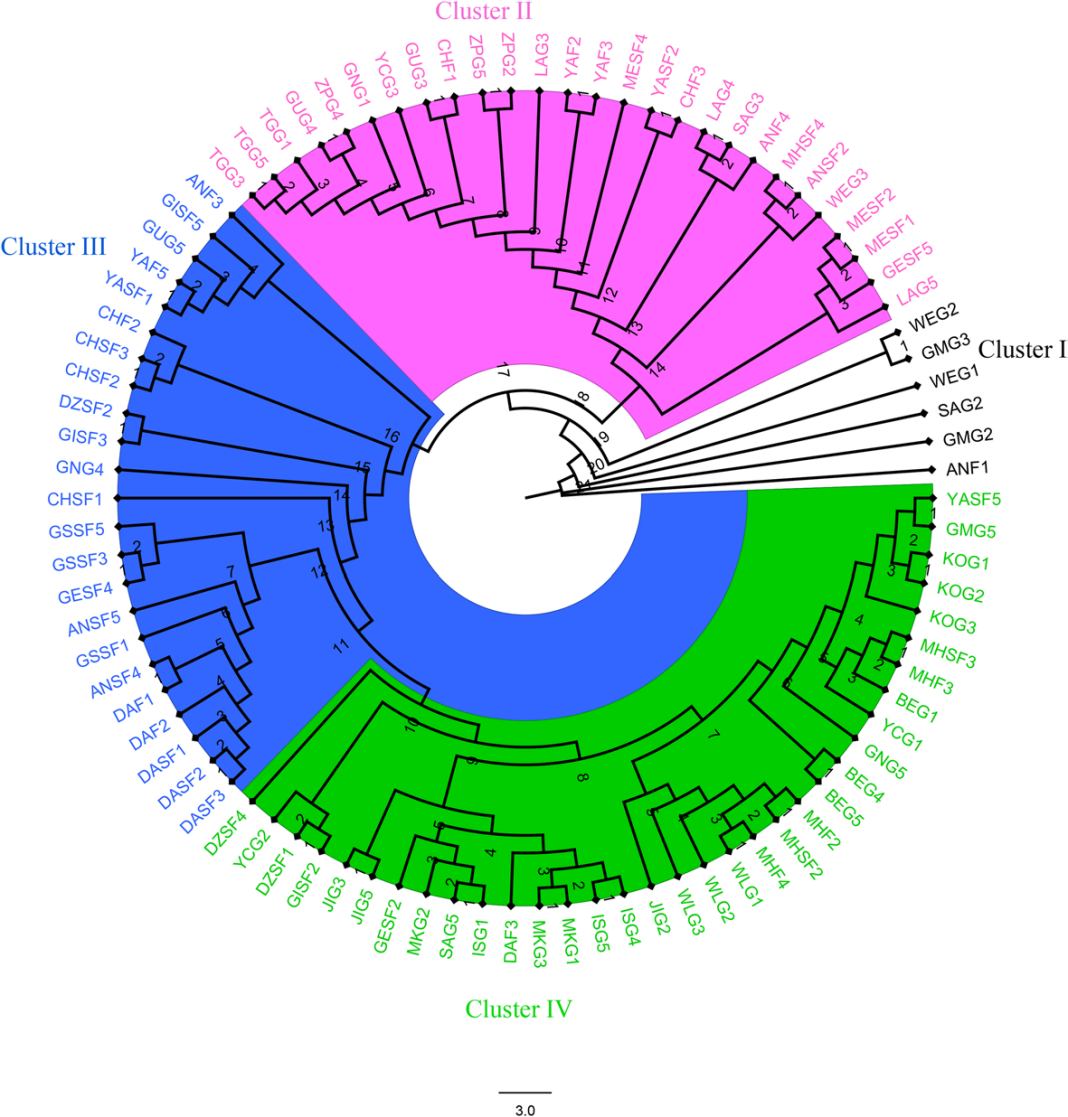
Maximum-likelihood (ML) phylogeny of *C. arabica* L. accessions inferred from RAxML using SNPs detected in whole-genome resequencing data. The accessions within different clades are highlighted with different colors

We inferred the phylogenetic relationships of the 90 accessions of *C. arabica* L. by constructing a Maximum-likelihood phylogenetic tree using the whole-genome SNP markers. The analysis revealed four major clusters, and each cluster also sub-divided into sub-clades. Forest-based accessions sampled from southwest Ethiopia were distributed in different clusters, while those sampled from the southeast part clustered together except MHSF4. Cluster I (Black), contained six accessions, of which two were collected from the northwest, while four were collected from the southwest. Cluster II (Purple) consisted of accessions collected from the southwest and the north part of the country. Cluster III (Blue) mainly consisted of accessions sampled from the southwest parts of the country i.e., GUG5, YCG2 and GNG4 sampled from the country’s north, south and southeastern parts, clustered together. Almost all of the south and southeast accession were clustered in group IV (Green). The garden coffee accessions collected from similar geographical areas consistently clustered together in the same group (e.g., TGG1, TGG3 and TGG5; ZPG2, ZPG4 and ZPG5; LAG3, LAG4 and LAG5; KOG1, KOG2 and KOG3; BEG1, BEG4 and BEG5; WLG1, WLG2 and WLG3; JIG2, JIG3 and JIG5; MKG1, MKG2 and MKG3, and ISG1, ISG2 and ISG3). Only, GNG, GUG, YCG, SAG, GMG and WEG garden coffee samples showed exceptional grouping patterns (Fig. 3). This suggests that the SNP markers identified in this study have the potential to give insights into the evolutionary relationship of the coffee accessions.

## Discussion

### Whole-genome sequence diversity

Presently, whole-genome resequencing is the most convenient approach for genome-wide SNP identification. It provides information of great relevance for crop genetics and breeding [35]. Besides, characterization of genome-wide DNA variation can help to understand the trait-genotype associations [37, 47]. In this study, we performed the whole-genome resequencing analysis of coffee, in which diverse accessions sampled from different geographic regions and production systems were included. The percentage of reads mapped onto the reference genome varied from 88.93 to 98.11% indicating the good quality of the data generated. The observed variation in the mapping rate could be attributed to the difference between the sequenced accessions and the reference genome. SNP and InDels identification using the *C. arabica* L. nuclear, mitochondrial and chloroplast genomes yielded a total of 9.74 million SNPs and 1.34 million InDels across 90 accessions. These variants were heterogeneously distributed across the eleven chromosomes of *C. arabica* L. Such uneven dispersal of variants has also been reported in other plant species such as in *Solanum melongena* and *C. canephora* [35, 48].

The variant identified by the resequencing study could be used for various studies including marker-assisted selection, genome-wide association studies, phylogenetic and diversity analyses [49]. SNPs also considered a valuable genetic marker, and often associated with the gene or trait of interest. Because of this, they are widely used genetic markers to identify genes responsible for traits of agricultural importance [33, 36]. A genome-wide association study was performed in coffee to identify genomic regions associated with lipid, cafestol and kahweol contents in green coffee beans [50]. The study discovered SNP located inside or near candidate genes related to metabolic pathways of these chemical compounds. SNP markers have also been utilized in coffee genetic diversity and population structure analysis [49]. In other studies, SNP markers associated with pathways of caffeine and trigonelline biosynthesis were reported [50]. Further, Tran et al. [51] identified 1444 non-synonymous SNPs associated with caffeine content using the draft genome of *C. arabica* L. Among these, the Kyoto Encyclopedia of Genes and Genome pathways analysis discovered 11 SNPs that have direct associations with genes encoding enzymes involved in caffeine biosynthesis pathways. These studies demonstrated the importance of SNP in identifying the genetic basis of traits of interest. The resequencing efforts of [52], identified SNPs that are potentially responsible for bacterial wilt disease in the *Capsicum annuum.* Furthermore, [53] highlighted that whole-genome resequencing is critical for the development of molecular markers. In this regard, marker development from variants identified from sequencing data has been done successfully for various agronomical important traits [54]. Thus, the SNP identified in the present study could be a valuable source of new allelic variations to advance coffee genomic research and germplasm improvement programs. Further, analyzing the diversity in the coffee genome could help uncover variants that could be used to better understand the genetic basis of agriculturally important traits.

### SNP analysis

Phenotypic variations in crop plants are the result of variation at the DNA level [55]. SNP is an abundant form of genetic variation and may cause phenotypic diversity among individuals [36]. Based on the nucleotide substitution SNP is generally classified as transitions and transversions [56]. In this study, the number of SNP with transitions was two-fold higher than SNP with transversions. G/A and C/T transitions were observed in equal numbers. The transition/transversion ratio was 1.97, which indicates transitions were the most frequent mutations similar to the findings from previous reports. For instance, [57, 58] reported a Ts/Tv ratio of 2.0 and 2.4 in rice, respectively. Generally, transversion SNP mutations have a high potential to alter the amino acid sequence of proteins than transitions [59]. Our study indicated that most of the detected SNP were located in intergenic and non-coding regions of the coffee genome. This suggests that these SNPs do not affect the gene functions. Similar results have also been reported in the previous studies on coffee, tea, potato and rice [31, 38, 59, 60].

The variant effects that might affect the protein-coding sequences include: synonymous/non-synonymous amino acid replacement, start codon gains or losses, stop codon gains or losses [45]. In this study, only 0.11% of SNP with high impact effects on genes were detected in the coffee genome. These SNPs might disturb the proper functioning of genes ultimately affecting the enzyme activity. Generally, the non-synonymous SNP in the coding sequence are disruptive and result in gene product change [36, 59, 60]. Hence, the identification of SNP found in genes, and analysis of their effects on phenotype could help to understand their impact on gene function and contribute to crop improvement programs [36]. In a previous study, stop gain, splice donor variant, intron variant, and splice acceptor variant were reported as disruptive variant effects in coffee affecting the proper functioning of genes [31]. Hence, the determination of the genomic location of variants contributes to identification of the genetic region responsible for trait variations.

### Phylogenetic analysis

SNP markers have been employed for analyzing the phylogenetic relationships and differences between genotypes [61]. In this study, the phylogenetic relationship of the 90 coffee accessions was analyzed using whole-genome SNP markers. The analysis revealed four major groups, comprising several sub-clades. Clusters I, II and III mainly contained accessions from the southwest. It is also noted that coffee accessions collected from southwest Ethiopia were found in different clusters. Specifically, the grouping of accessions collected from the southwest regions in different groups could explain coffee accessions that originated in that region had a broad genetic base. In another study, Spinoso-Castilillo et al. [11] reported that the SNP markers generated by DArTseq technology separated the 87 accessions of *Coffea* spp. into five distinct groups. Further, Silvestrini et al. [62] reported that even if coffee accessions had originated in the same localities, there was a possibility of separating genetically by the domestication process due to human selection activity. The finding also supports earlier reports that suggested southwest Ethiopia as the center of origin and diversity of *C. arabica* L. [8, 12, 17, 63].

Most of the garden coffee accessions collected from the north parts of the country are grouped with southwest forest-based accessions. Whereas, the majority of garden coffee accessions sampled from southern parts of the country are grouped with southeast forest-based accession. Delsuc et al. [64] reported that phylogenetic analysis is one of the tools that could help understand the evolutionary relationships of crop plants. Hence, the grouping of garden coffee accessions sampled from different areas with southwest and southeast forest-based accessions shows the ancestor of these accessions probably originated in the southwest and southeast. The garden coffee accessions collected from similar geographical areas consistently clustered together in the same group. These accessions also formed a sub-clade within a forest-based accession clade.

A recent study by Benti et al. [12] on the Ethiopian commercial *C. arabica* L. varieties also found the grouping of varieties into different clusters regardless of their geographic origin. The clustering pattern found in this study could indicate the presence of a high level of genetic diversity within coffee accessions sampled from the same geographic origin. Furthermore, the garden coffee accessions sampled from the neighboring regions were consistently grouped with few exceptions (Fig. 3). This might have been attributed to gene flow between adjacent populations or the garden coffee farms probably established from seeds obtained from the same source. The clustering of southwest coffee accessions with the south and southeast population was previously reported [8, 65, 66]. Moreover, Mishra et al. [67] noted that coffee accessions collected from the same geographical origin in Ethiopia did not cluster together. All these studies indicate that the *C. arabica* L. germplasm found in Ethiopia has a broad genetic base, and is valuable in developing varieties that could sustain global coffee production.

## Conclusion

The availability of reference genomes and the continuous improvements of genetic data analysis methods are fostering resequencing studies. In this study, we performed the resequencing of coffee accessions, which has allowed the identification of genetic markers, such as SNPs and InDels. The SNPs discovered in this study might contribute to the variation in important pathways of genes for important agronomic traits such as caffeine content, yield, disease, and pest in coffee. Moreover, the genome resequencing data and the genetic markers identified from 90 accessions provide insight into the genetic variation of the coffee germplasm and facilitate a broad range of genetic studies.

## Supplementary Information

**Additional file 1: Table S1**. The details of the analyzed 90 accession of *C. arabica* L.

**Additional file 2: Table S2**. Summary of the *C. arabica* L. whole-genome sequencing data.

**Additional file 3: Table S3**. Amino acid changes identified by SnpEff software. Rows are reference amino acids and columns are changed amino acids. E.g., Row 'A' column 'E' indicates how many 'A' amino acids have been replaced by 'E' amino acids.

**Additional file 4: Table S4**. Summary count of SNPs with effects on the genome.

## Data Availability

The data generated and analyzed during this study are included in this article, its supplemental information files and the sequence data have been deposited into NCBI sequence read archive under submission identifier SUB10821951 with the Bio project identifier PRJNA790687.

## Declarations

Study protocol must comply with relevant institutional, national, and international guidelines and legislation.
