# Supplementary material for: Whole-genome resequencing of Coffea arabica L. (Rubiaceae) genotypes identify SNP and unravels distinct groups showing a strong geographical pattern
Source: BMC Plant Biol. 2022 Feb 14;22:69. doi: 10.1186/s12870-022-03449-4 (PMC8842891; doi:10.1186/s12870-022-03449-4)
Supplement: Supplementary file 1 — Additional file 1: Table S1. The details of the analyzed 90 accession of C. arabica L. [file 12870_2022_3449_MOESM1_ESM.doc]

**Additional file 1: Table S1.** The details of the analyzed 90 accession of *C. arabica* L.

| **S.N.** | **Accession number** | **Accession**  **code** | **Place of collections** | | | | | **Sampling**  **site** | **Altitude** |
| --- | --- | --- | --- | --- | --- | --- | --- | --- | --- |
|  |  |  | **Region/Province** | | | **District** | |  |  |
| 1 | 212430 | GUG3 | Amhara | | | Gungua | | Garden | 1680 |
| 2 | 212426 | GUG4 | Amhara | | | Gungua | | Garden | 1680 |
| 3 | 212420 | GUG5 | Amhara | | | Gungua | | Garden | 1680 |
| 4 | 8739 | SAG2 | Oromia | | | Sasiga | | Garden | 1837 |
| 5 | 8731 | SAG3 | Oromia | | | Sasiga | | Garden | 1579 |
| 6 | 8713 | SAG5 | Oromia | | | Sasiga | | Garden | 1711 |
| 7 | 244310 | WLG1 | Amhara | | | Kalu | | Garden | 1520 |
| 8 | 244309 | WLG2 | Amhara | | | Kalu | | Garden | 1770 |
| 9 | 244301 | WLG3 | Amhara | | | Kalu | | Garden | 2170 |
| 10 | 8784 | ISG1 | SNNP | | | Isara-Tocha | | Garden | 2038 |
| 11 | 8772 | ISG4 | SNNP | | | Isara-Tocha | | Garden | 2020 |
| 12 | 8768 | ISG5 | SNNP | | | Isara-Tocha | | Garden | 2036 |
| 13 | 8700 | MKG1 | SNNP | | | Melokoza | | Garden | 1700 |
| 14 | 8698 | MKG2 | SNNP | | | Melokoza | | Garden | 1750 |
| 15 | 8692 | MKG3 | SNNP | | | Melokoza | | Garden | 1500 |
| 16 | 212005 | ZPG2 | Amhara | | | Bahrdarzuria | | Garden | - |
| 17 | 242651 | ZPG4 | Amhara | | | Bahrdarzuria | | Garden | 1870 |
| 18 | 242649 | ZPG5 | Amhara | | | Bahrdarzuria | | Garden | 1850 |
| 19 | 8669 | JIG2 | SNNP | | | Jinka | | Garden | 1400 |
| 20 | 8668 | JIG3 | SNNP | | | Jinka | | Garden | 1370 |
| 21 | 8660 | JIG5 | SNNP | | | Jinka | | Garden | 1360 |
| 22 | 23454 | GNG1 | Oromia | | | Giner | | Garden | 1510 |
| 23 | 23449 | GNG4 | Oromia | | | Giner | | Garden | 1510 |
| 24 | 23444 | GNG5 | Oromia | | | Giner | | Garden | 1510 |
| 25 | 8919 | BEG1 | Oromia | | | Berbere | | Garden | 1680 |
| 26 | 8911 | BEG4 | Oromia | | | Berbere | | Garden | 1600 |
| 27 | 8901 | BEG5 | Oromia | | | Berbere | | Garden | 1660 |
| 28 | 218900 | LAG3 | Oromia | | | Lalo-Asabi | | Garden | 1600 |
| 29 | 218898 | LAG4 | Oromia | | | Lalo-Asabi | | Garden | 1720 |
| 30 | 218895 | LAG5 | Oromia | | | Lalo-Asabi | | Garden | 1720 |
| 31 | 218809 | GMG2 | Oromia | | | Gimbi | | Garden | 1760 |
| 32 | 218806 | GMG3 | Oromia | | | Gimbi | | Garden | 1710 |
| 33 | 212391 | GMG5 | Oromia | | | Gimbi | | Garden | 1900 |
| 34 | 8764 | WEG1 | Benishangule-Gumuz | | | Wenbera | | Garden | 2373 |
| 35 | 8731 | WEG2 | Benishangule-Gumuz | | | Wenbera | | Garden | 1579 |
| 36 | 8759 | WEG3 | Benishangule-Gumuz | | | Wenbera | | Garden | 1755 |
| 37 | 211949 | YCG1 | SNNP | | | Y/Chefe | | Garden | 1880 |
| 38 | 211948 | YCG2 | SNNP | | | Y/Chefe | | Garden | 1880 |
| 39 | 24910 | YCG3 | SNNP | | | | Y/Chefe | Garden | 2030 |
| 40 | 24877 | KOG1 | SNNP | | | | Kochere | Garden | 1720 |
| 41 | 24885 | KOG2 | SNNP | | | | Kochere | Garden | 1720 |
| 42 | 24888 | KOG3 | SNNP | | | | Kochere | Garden | 1752 |
| 43 | 244254 | TGG1 | Tigray | | | | Abergele | Garden | 1910 |
| 44 | 244271 | TGG3 | Tigray | | | | Tselemti | Garden | 1400 |
| 45 | 244274 | TGG5 | Tigray | | | | Laelay Adiyabo | Garden | 1800 |
| 46 | 21891 | ANF1 | Oromia | | | | Anfilo | Forest-based | 1300 |
| 47 | 21861 | ANF3 | Oromia | | | | Anfilo | Forest-based | 1820 |
| 48 | 21859 | ANF4 | Oromia | | | | Anfilo | Forest-based | 1820 |
| 49 | 22986 | CHF1 | Oromia | | | | Chora | Forest-based | 1910 |
| 50 | 22985 | CHF2 | Oromia | | | | Chora | Forest-based | 1910 |
| 51 | 22974 | CHF3 | Oromia | | | | Chora | Forest-based | 1620 |
| 52 | 212106 | DAF1 | Oromia | | | | Darimu | Forest-based | 1540 |
| 53 | 212100 | DAF2 | Oromia | | | | Darimu | Forest-based | 1500 |
| 54 | 212080 | DAF3 | Oromia | | | | Darimu | Forest-based | 1600 |
| 55 | 8844 | MHF2 | Oromia | | | | M/Harena-Buluk | Forest-based | 1680 |
| 56 | 8841 | MHF3 | Oromia | | | | M/Harena-Buluk | Forest-based | 1680 |
| 57 | 8835 | MHF4 | Oromia | | | | M/Harena-Buluk | Forest-based | 1650 |
| 58 | 21632 | YAF2 | Oromia | | | | Yayo | Forest-based | 1480 |
| 59 | 21630 | YAF3 | Oromia | | | | Yayo | Forest-based | 1480 |
| 60 | 21628 | YAF5 | Oromia | | | | Yayo | Forest-based | 1480 |
| 61 | 21818 | ANSF2 | Oromia | | | | Anfilo | Forest-based | 1450 |
| 62 | 21814 | ANSF4 | Oromia | | | | Anfilo | Forest-based | 1460 |
| 63 | 21810 | ANSF5 | Oromia | | | | Anfilo | Forest-based | 1460 |
| 64 | 230462 | CHSF1 | Oromia | | | | Chora | Forest-based | 1940 |
| 65 | 22904 | CHSF2 | Oromia | | | | Chora | Forest-based | 1940 |
| 66 | 22901 | CHSF3 | Oromia | | | | Chora | Forest-based | 1940 |
| 67 | 212092 | DASF1 | Oromia | | | | Darimu | Forest-based | 1530 |
| 68 | 212090 | DASF2 | Oromia | | | | Darimu | Forest-based | 1530 |
| 69 | 212089 | DASF3 | Oromia | | | | Darimu | Forest-based | 1530 |
| 70 | 23435 | MHSF2 | Oromia | | | | M/Harena-Buluk | Forest-based | 1560 |
| 71 | 23434 | MHSF3 | Oromia | | | | M/Harena-Buluk | Forest-based | 1560 |
| 72 | 23429 | MHSF4 | Oromia | M/Harena-Buluk | | | | Forest-based | 1560 |
| 73 | 21662 | YASF1 | Oromia | Yayo | | | | Forest-based | 1640 |
| 74 | 21661 | YASF2 | Oromia | Yayo | | | | Forest-based | 1640 |
| 75 | 21648 | YASF5 | Oromia | Yayo | | | | Forest-based | 1640 |
| 76 | 219391 | DZSF1 | SNNP | | Dizu | | | Forest-based | - |
| 77 | 212047 | DZSF2 | SNNP | | Dizu | | | Forest-based | - |
| 78 | 212029 | DZSF4 | SNNP | | Dizu | | | Forest-based | - |
| 79 | 211935 | GESF2 | Oromia | | Gera | | | Forest-based | - |
| 80 | 211931 | GESF4 | Oromia | | Gera | | | Forest-based | - |
| 81 | 212336 | GESF5 | Oromia | | Gera | | | Forest-based | - |
| 82 | 219412 | GISF2 | SNNP | | Ginbo | | | Forest-based | - |
| 83 | 219408 | GISF3 | SNNP | | Ginbo | | | Forest-based | - |
| 84 | 219401 | GISF5 | SNNP | | Ginbo | | | Forest-based | - |
| 85 | 23182 | MESF1 | Oromia | | Metu | | | Forest-based | 1570 |
| 86 | 23178 | MESF2 | Oromia | | Metu | | | Forest-based | 1570 |
| 87 | 23175 | MESF4 | Oromia | | Metu | | | Forest-based | 1570 |
| 88 | 212255 | GSSF1 | SNNP | | Gesha | | | Forest-based | 1930 |
| 89 | 212249 | GSSF3 | SNNP | | Gesha | | | Forest-based | 1940 |
| 90 | 212227 | GSSF5 | SNNP | | Gesha | | | Forest-based | 1650 |
