# Supplementary material for: Whole-genome resequencing of Coffea arabica L. (Rubiaceae) genotypes identify SNP and unravels distinct groups showing a strong geographical pattern
Source: BMC Plant Biol. 2022 Feb 14;22:69. doi: 10.1186/s12870-022-03449-4 (PMC8842891; doi:10.1186/s12870-022-03449-4)
Supplement: Supplementary file 2 — Additional file 2: Table S2. Summary of the C. arabica L. whole-genome sequencing data. [file 12870_2022_3449_MOESM2_ESM.doc]

**Additional file 2: Table S2.** Summary of the *C. arabica* L. whole-genome sequencing data.

| **Accession**  **code** | **Total reads** | **Mapped reads** | **Percent mapped (%)** | **Paired read** | **Ratio** | **GC%** |
| --- | --- | --- | --- | --- | --- | --- |
| ANF1 | 95235925 | 92933700 | 97.58% | 89621894 | 94.59% | 37.71 |
| ANF3 | 69761896 | 67731720 | 97.09% | 65259088 | 94.02% | 37.73 |
| ANF4 | 72795524 | 70979996 | 97.51% | 68022484 | 93.92% | 37.49 |
| ANSF2 | 77812606 | 74976044 | 96.35% | 71886286 | 92.86% | 37.47 |
| ANSF4 | 79654427 | 77693944 | 97.54% | 74949640 | 94.57% | 37.54 |
| ANSF5 | 78381015 | 76386790 | 97.46% | 73716354 | 94.54% | 37.49 |
| BEG1 | 75788636 | 74171338 | 97.87% | 71631360 | 94.94% | 37.32 |
| BEG4 | 78009411 | 76403121 | 97.94% | 73721502 | 94.94% | 37.49 |
| BEG5 | 73872980 | 72479604 | 98.11% | 69950752 | 95.14% | 37.47 |
| CHF1 | 73331004 | 71635505 | 97.69% | 68786902 | 94.28% | 37.55 |
| CHF2 | 78902052 | 77128375 | 97.75% | 74132410 | 94.43% | 37.41 |
| CHF3 | 80331170 | 78571146 | 97.81% | 75493578 | 94.45% | 37.53 |
| CHSF1 | 75590205 | 73883394 | 97.74% | 71004912 | 94.43% | 37.41 |
| CHSF2 | 68511935 | 67069054 | 97.89% | 64315992 | 94.36% | 37.4 |
| CHSF3 | 82346876 | 80140813 | 97.32% | 77097634 | 94.11% | 37.53 |
| DAF1 | 72299766 | 70212595 | 97.11% | 67454500 | 93.76% | 37.67 |
| DAF2 | 77901273 | 75874967 | 97.40% | 72731830 | 93.86% | 37.47 |
| DAF3 | 69288094 | 67483303 | 97.40% | 65030752 | 94.31% | 37.54 |
| DASF1 | 71576269 | 69633079 | 97.29% | 67040642 | 94.17% | 37.61 |
| DASF2 | 71878980 | 69956591 | 97.33% | 67422052 | 94.29% | 37.58 |
| DASF3 | 93252374 | 90869019 | 97.44% | 87757362 | 94.61% | 37.53 |
| DZSF1 | 72931860 | 70517096 | 96.69% | 67678926 | 93.27% | 37.55 |
| DZSF2 | 70177728 | 68367164 | 97.42% | 65765326 | 94.20% | 37.45 |
| DZSF4 | 74808004 | 72705139 | 97.19% | 69935710 | 93.98% | 37.52 |
| GESF2 | 79959277 | 78295175 | 97.92% | 75206038 | 94.54% | 37.38 |
| GESF4 | 80094032 | 77482810 | 96.74% | 74083394 | 93.04% | 37.54 |
| GESF5 | 76221939 | 74226569 | 97.38% | 71402122 | 94.15% | 37.58 |
| GISF2 | 87927802 | 85511879 | 97.25% | 82130758 | 93.90% | 37.48 |
| GISF3 | 78824289 | 76666584 | 97.26% | 73920232 | 94.28% | 37.52 |
| GISF5 | 86080272 | 84318645 | 97.95% | 80913680 | 94.50% | 37.29 |
| GMG2 | 66362418 | 64795517 | 97.64% | 62490602 | 94.64% | 37.44 |
| GMG3 | 69994455 | 68071732 | 97.25% | 65536882 | 94.10% | 37.45 |
| GMG5 | 72451997 | 70483913 | 97.28% | 67975434 | 94.27% | 37.57 |
| GNG1 | 85788748 | 83731875 | 97.60% | 80281996 | 94.10% | 37.33 |
| GNG4 | 73897868 | 72244267 | 97.76% | 69524870 | 94.56% | 37.26 |
| GNG5 | 65131994 | 63295289 | 97.18% | 61060674 | 94.19% | 37.18 |
| GSSF1 | 75283753 | 73853059 | 98.10% | 71327714 | 95.25% | 37.44 |
| GSSF3 | 86912974 | 84410512 | 97.12% | 80674406 | 93.33% | 37.39 |
| GSSF5 | 77591847 | 75532729 | 97.35% | 72534846 | 93.96% | 37.57 |
| GUG3 | 74672315 | 68381870 | 91.58% | 64460372 | 86.89% | 37.4 |
| GUG4 | 65934395 | 60461473 | 91.70% | 57272336 | 87.38% | 37.31 |
| GUG5 | 87474767 | 78591962 | 89.85% | 73970854 | 85.11% | 37.5 |
| ISG1 | 77069234 | 75147443 | 97.51% | 72297832 | 94.28% | 37.3 |
| ISG4 | 76307869 | 74438157 | 97.55% | 71640020 | 94.36% | 37.32 |
| ISG5 | 69465691 | 67964613 | 97.84% | 65036772 | 94.10% | 37.26 |
| JIG2 | 72375699 | 70754199 | 97.76% | 67764616 | 94.12% | 37.37 |
| JIG3 | 75034105 | 73390515 | 97.81% | 70529406 | 94.47% | 37.4 |
| JIG5 | 82427826 | 80803776 | 98.03% | 77661286 | 94.71% | 37.22 |
| KOG1 | 71477666 | 69772193 | 97.61% | 67367026 | 94.70% | 37.58 |
| KOG2 | 69609024 | 67870334 | 97.50% | 65358576 | 94.37% | 37.47 |
| KOG3 | 88319831 | 86329467 | 97.75% | 83308600 | 94.77% | 37.53 |
| LAG3 | 78983139 | 77235225 | 97.79% | 74418968 | 94.70% | 37.38 |
| LAG4 | 67796074 | 66136327 | 97.55% | 63458914 | 94.08% | 37.49 |
| LAG5 | 79761292 | 77479901 | 97.14% | 74287932 | 93.62% | 37.39 |
| MESF1 | 82571095 | 79830667 | 96.68% | 76450170 | 93.08% | 37.41 |
| MESF2 | 85512338 | 83000443 | 97.06% | 80008046 | 94.04% | 37.46 |
| MESF4 | 79604946 | 76663087 | 96.30% | 73234568 | 92.51% | 37.55 |
| MHF2 | 68228579 | 66430237 | 97.36% | 64028212 | 94.27% | 38 |
| MHF3 | 73778056 | 71691532 | 97.17% | 69040976 | 94.03% | 37.64 |
| MHF4 | 78633917 | 76615247 | 97.43% | 73943554 | 94.48% | 37.62 |
| MHSF2 | 79098965 | 77305151 | 97.73% | 74585722 | 94.76% | 37.63 |
| MHSF3 | 83951034 | 82034333 | 97.72% | 79240718 | 94.84% | 37.48 |
| MHSF4 | 73862856 | 69522772 | 94.12% | 66221678 | 90.16% | 38.06 |
| MKG1 | 76799280 | 75321202 | 98.08% | 72083974 | 94.34% | 37.24 |
| MKG2 | 85198228 | 78372066 | 91.99% | 73903276 | 87.35% | 37.34 |
| MKG3 | 80358339 | 74953626 | 93.27% | 70819470 | 88.71% | 37.34 |
| SAG2 | 71353991 | 65594043 | 91.93% | 61990842 | 87.41% | 37.88 |
| SAG3 | 67860579 | 60351687 | 88.93% | 56508708 | 83.87% | 37.54 |
| SAG5 | 89034029 | 86965041 | 97.68% | 83283800 | 94.01% | 37.19 |
| TGG1 | 68721511 | 67029159 | 97.54% | 64423626 | 94.24% | 37.49 |
| TGG3 | 68020850 | 66114402 | 97.20% | 63326448 | 93.63% | 37.69 |
| TGG5 | 66871342 | 65078445 | 97.32% | 62610758 | 94.12% | 37.47 |
| WEG1 | 69665853 | 68087169 | 97.73% | 65684070 | 94.76% | 37.36 |
| WEG2 | 68642023 | 66635602 | 97.08% | 64218894 | 94.02% | 37.51 |
| WEG3 | 65905295 | 64165175 | 97.36% | 61649432 | 94.03% | 37.52 |
| WLG1 | 75416628 | 69777164 | 92.52% | 66101914 | 88.19% | 37.39 |
| WLG2 | 81274776 | 79648975 | 98.00% | 76896306 | 95.03% | 37.45 |
| WLG3 | 74052342 | 72586236 | 98.02% | 69921046 | 94.88% | 37.4 |
| YAF2 | 66598336 | 65110057 | 97.77% | 62831782 | 94.81% | 37.41 |
| YAF3 | 70220862 | 68577582 | 97.66% | 66111886 | 94.62% | 37.65 |
| YAF5 | 87353631 | 85108488 | 97.43% | 81778418 | 94.10% | 37.52 |
| YASF1 | 81379713 | 78916059 | 96.97% | 75521844 | 93.28% | 37.46 |
| YASF2 | 80705637 | 78135209 | 96.82% | 75113510 | 93.54% | 37.49 |
| YASF5 | 74910439 | 73043617 | 97.51% | 70143696 | 94.09% | 37.75 |
| YCG1 | 70713760 | 68636579 | 97.06% | 66096392 | 93.93% | 37.7 |
| YCG2 | 89235817 | 86913375 | 97.40% | 83480824 | 94.03% | 37.59 |
| YCG3 | 75033764 | 73304114 | 97.69% | 70433990 | 94.36% | 37.44 |
| ZPG2 | 90216484 | 88056344 | 97.61% | 84307334 | 93.93% | 37.46 |
| ZPG4 | 78651692 | 76661475 | 97.47% | 73261102 | 93.66% | 37.3 |
| ZPG5  **Total**  **Mean** | 73816372  **6886925961**  **76521399** | 72202309  **6673920176**  **74154668** | 97.81%  -  **97%** | 69382718  **6406944750**  **71188275** | 94.47%  -  **93.51%** | 37.34  -  **37.48** |
