# Supplementary material for: Whole-genome resequencing of Coffea arabica L. (Rubiaceae) genotypes identify SNP and unravels distinct groups showing a strong geographical pattern
Source: BMC Plant Biol. 2022 Feb 14;22:69. doi: 10.1186/s12870-022-03449-4 (PMC8842891; doi:10.1186/s12870-022-03449-4)
Supplement: Supplementary file 4 — Additional file 4: Table S4. Summary count of SNPs with effects on the genome. [file 12870_2022_3449_MOESM4_ESM.doc]

**Additional file 4: Table S4.** Summary count of SNPs with effects on the genome.

4.1 Number of effects by type

| **Type (alphabetical order)** | | | | | | | | **Count** | | | **Percent** | **Category** |
| --- | --- | --- | --- | --- | --- | --- | --- | --- | --- | --- | --- | --- |
| 3_prime_UTR_variant | | | | | | | | 184,709 | | | 0.573% | Modifier |
| 5_prime_UTR_premature_start_codon_gain_variant | | | | | | | | 15,197 | | | 0.047% | Low |
| 5_prime_UTR_variant | | | | | | | | 140,468 | | | 0.436% | Modifier |
| downstream_gene_variant | | | | | | | | 8,275,347 | | | 25.680% | Modifier |
| initiator_codon_variant | | | | | | | | 88 | | | 0.000% | Low |
| intergenic_region | | | | | | | | 9367280 | | | 29.069% | Modifier |
| intragenic_variant | | | | | | | | 83,876 | | | 0.260% | Modifier |
| intron_variant | | | | | | | | 1,966,285 | | | 6.102% | Modifier |
| missense_variant | | | | | | | | 359,187 | | | 1.115% | Moderate |
| non_coding_transcript_variant | | | | | | | | 187,053 | | | 0.580% | Modifier |
| splice_acceptor_variant | | | | | | | | 3,694 | | | 0.011% | High |
| splice_donor_variant | | | | | | | | 3,332 | | | 0.010% | High |
| splice_region_variant | | | | | | | | 46,685 | | | 0.145% | High |
| start_lost | | | | | | | | 817 | | | 0.003% | High |
| stop_gained | | | | | | | | 14,625 | | | 0.045% | High |
| stop_lost | | | | | | | | 1,123 | | | 0.003% | High |
| stop_retained_variant | | | | | | | | 306 | | | 0.001% | Low |
| synonymous_variant | | | | | | | | 215,712 | | | 0.669% | Low |
| upstream_gene_variant | | | | | | | | 8,567,930 | | | 26.588% | Modifier |
| 4.2 Base changes | | | |  | | | |  | | |  |  |
|  | | **A** | **C** | | **G** | | **T** | | |  | | |
| **A** | | 0 | 338561 | | 1255604 | | 490953 | | |  | | |
| **C** | | 648472 | 0 | | 272404 | | 1866931 | | |  | | |
| G | | 1863552 | 271469 | | 0 | | 648259 | | |  | | |
| T | | 491097 | 1256788 | | 339714 | | 0 | | |  | | |
|  | | | |  | | | |  | | |  |  |
|  | 4.3 Ts/Tv (transitions/transversions) ratio | | | | |  | |  |  |  |  | |
|  | Transitions | | | | | 204,665,968 | |  |  |  |  | |
|  | Transversions | | | | | 103,693,961 | |  |  |  |  | |
|  | Ts/Tv ratio | | | | | 1.9738 | |  |  |  |  | |
